# Validation of the Toronto Empathy Questionnaire (TEQ) Among Medical Students in China: Analyses Using Three Psychometric Methods

**DOI:** 10.3389/fpsyg.2020.00810

**Published:** 2020-04-28

**Authors:** Richard Huan Xu, Eliza Lai-yi Wong, Sabrina Yu-jun Lu, Ling-ming Zhou, Jing-hui Chang, Dong Wang

**Affiliations:** ^1^Jockey Club School of Public Health and Primary Care, The Chinese University of Hong Kong, Hong Kong, China; ^2^Faculty of Education, The Chinese University of Hong Kong, Hong Kong, China; ^3^School of Health Management, Southern Medical University, Guangzhou, China

**Keywords:** empathy, medical student, China, validation, Rasch model, item response theory, classical test theory

## Abstract

This study aimed to validate the simplified Chinese version of the Toronto Empathy Questionnaire (cTEQ) for use with the Chinese population. The original English version of the TEQ was translated into simplified Chinese based on international criteria. Psychometric analyses were performed based on three psychometric methods: classical test theory (CTT), item response theory (IRT), and Rasch model theory (RMT). Differential item functioning analysis was adopted to check possible item bias caused by responses from different subgroups based on sex and ethnicity. A total of 1296 medical students successfully completed the TEQ through an online survey; 75.2% of respondents were female and the average age was 19 years old. Forty students completed the questionnaire 2 weeks later to assess the test–retest reliability of the questionnaire. Confirmatory factor analysis supported a 3-factor structure of the cTEQ. The CTT analyses confirmed that the cTEQ has sound psychometric properties. However, IRT and RMT analyses suggested some items might need further modifications and revisions.

## Introduction

Although empathy has mainly been studied, both theoretically and conceptually, in the sphere of philosophy, psychology, and ethics, using different concepts and constructs, in recent decades, an increasing number of studies have reported identifying the role of empathy in the context of medical, nursing, and other healthcare professions in improving the quality of healthcare services (Brockhouse et al., 2011; Smajdor et al., 2011; Cunico et al., 2012). The importance of empathy is highlighted by a number of studies that propose that it should be considered as one of the key elements involved in medical education programs (Spreng et al., 2009; Smajdor et al., 2011; Kourmousi et al., 2017).

The word empathy was based on a translation of the German word “*Einfühlung*,” which means “feeling into” (Wispé, 1986). Generally speaking, it refers to the ability to understand and share the feelings of others. Although empathy is not a new concept and its importance has been studied in a number of different fields, debate has never been resolved on the difference between the two main components of the construct of empathy: cognitive and emotional (Preston and de Waal, 2002). Different perspectives on empathy provide different understandings of this concept. Generally, emotional empathy implies an emotional reaction toward another person’s feelings (e.g., happiness), whereas it does not require understanding of why someone is expressing such feelings. On the other hand, cognitive empathy refers to the cognitive and intellectual apprehension of another person’s emotional state, although it does not necessarily entail experiencing the same emotional state. These two components have been used, either exclusively or interchangeably, to explain the concept of empathy in different studies. In the field of healthcare, Morse et al. (1992) further explained the theory of empathy, and showed that cognitive empathy describes when health professionals have the ability to intellectually understand a patients’ feelings from an objective perspective, whereas emotional empathy describes when professionals have the ability to experience and share their patients’ feelings.

Empathy has been identified as a key component of professionalism in medicine (Stern, 2006), and the most frequently mentioned personality attribute of the humanistic doctor (Linn et al., 1987). However, given that doctors and other health professionals do not have long term contact with patients, who often experience negative emotions, doctors/professionals are unlikely to provide psychological and social support for patients in an intense working environment. Cultivating empathy is difficult but important in medical practice. Some studies have found that medical students experience a decline in empathy for their patients during their medical training (Pohontsch et al., 2018). However, another study found that if doctors become too emotionally involved with their patients, they are highly likely to experience burnout (Kim, 2018). In conclusion, engagement of empathy in medical practice and training is essential to build a trusting doctor–patient relationship.

In China, the doctor–patient relationship has become quite intense in recent years. In 2014, more than 115 thousand cases were reported that were related to conflict in the doctor–patient relationship, and more than 66% of doctors indicated that they have experienced some kind of conflict with patients (Chinese Medical Doctor Association, 2015). Although reasons for conflict are varied and complicated, lack of empathy has been confirmed as one of the most important factors. With an increasing emphasis on empathy in medical practice, it is essential to have a reliable and valid instrument to measure empathy for doctors and associated healthcare professionals. A number of scales, developed based on different concepts and constructs (Spreng et al., 2009), have been introduced to evaluate empathy in different settings, for example, the Empathy Scale (Hogan, 1969), the Questionnaire Measure of Emotional Empathy (Mehrabian and Epstein, 1972), and the Interpersonal Reactivity Index (Davis, 1983). In healthcare settings, one of the most commonly used scales is the Jefferson Scale of Empathy (JSE), which has three versions (for medical students, professionals, and student professionals) and has been widely used in the United States and Europe to evaluate empathy in medical practice. In China, application of the JSE or other empathy scales in studies related to healthcare is highly insufficient. Based on our literature review, only one study, which investigated medical students from North China, reported the validity of a simplified Chinese version of the JSE in English literature and showed acceptable reliability (Wen et al., 2013). However, some concerns should be addressed. A minor concern is that the heterogeneity of reported psychometric properties of the JSE should be considered because of geographical and sociodemographic distinctions in China. The validity of the JSE should be further explored in different places in China. The major concern is, as Hojat et al. (2001) indicated, that the JSE is considered a measure of cognitive empathy; however, as already noted, empathy has, at the least, both cognitive and emotional dimensions, and a lack of measures of emotional empathy may limit the generalizability of the findings to some extent.

The Toronto empathy questionnaire (TEQ), an alternative measure developed by Spreng et al. (2009), is a unidimensional, brief, and valid instrument for the assessment of empathy. The TEQ was created to mainly assess empathy as an emotional process, but still captures variance associated with cognitive measures of empathy (Kourmousi et al., 2017). The TEQ developers aimed to create a measure to suitably assess an individual’s general capacity for empathy as a central process covering various levels, which other measures of empathy might fail to achieve because of their heterogeneity of concepts and constructs (Ickes, 1997). While the TEQ was not designed specifically for use within the healthcare professions, acceptable validity and reliability has been reported in different populations, including medical students (Youssef et al., 2014). However, its psychometric properties have seldom been investigated in non-English-speaking samples, and no independent validation studies of the TEQ have yet been conducted in China. The TEQ could be a useful measure to evaluate empathy from different perspectives in both China and other regions worldwide.

### Psychometric Methods

Given the great development of psychometric methods in the last several decades, it is challenging for researchers to choose a proper technique to assess the performance of a measure. In this paper, we introduce and compare the psychometric properties of the TEQ based on three main methods in psychometrics: classical test theory (CTT), item response theory (IRT), and Rasch model theory (RMT).

Classical test theory, founded by Spearman, is the most famous and traditional method used to assess the properties property of a measure. CTT analyses are performed on a test as an integrated whole rather than on the item level. CTT analyses assume that each person has an inherent attribute (true score), which is composed of the combination of the observed score and random error in a test measure. The error is normally distributed with a mean of 0 and an SD of 1. The smaller the error variance, the more accurately the true scores (inherent attributes) are reflected by the observed scores. Use of CTT has been ubiquitous (Crocker, 1986).

Item response theory is often referred to as latent trait analysis or modern test theory, and was introduced by Thurstone (1925). It was designed to assess the relationship between latent traits and their observed variables. IRT models establish a link between the properties of items of a measure, individual’s responses to the items of the measure, and the underlying trait being measured (Steinberg and Thissen, 2013). IRT theory assumes that the latent trait and the performance of the item can be organized along a continuum, and the main purpose of IRT is to establish the individual’s position on that continuum. There are some assumptions that have to be met before conducting IRT analysis. The details of IRT can be found in Linden and Hambleton (1997).

Rasch model theory is based on the work of Rasch (1993). Some scholars imply that the mathematical theory underlying RMT is a special case of IRT, while others disagree. RMT is prescriptive and requires the data to fit the model to generate invariant and interval level measures of items and persons simultaneously. The basic logic of RMT is that individuals always have a high probability of correctly answering easy questions but a low probability of correctly answering hard questions. In other words, the probability of an individual confirming an item is a logical function of the difference between the level of the person’s ability (to correctly answer that item) and the location of the item (the difficulty of the item) on an interval scale, which is then mapped on the same latent trait. The details of RMT are very widely known and can be found in many sources (Fischer and Molenaar, 1995; Bond, 2007; Christensen et al., 2013).

Because different methods usually adopt different techniques, produce different outcomes, and rely on different criteria to make a judgment, few studies have presented direct comparisons of them in the psychometric evaluation of one measurement. Given each method has its benefits and weaknesses, it is hard to say which one performs better than the others. Currently, CTT remains the dominant method in psychometric evaluation; however, the adoption of IRT and RMT is increasing. Therefore, in this study, we aimed to assess the reliability and validity of the Chinese version of the TEQ (cTEQ), and present and compare the results based on three different psychometric methods.

## Materials and Methods

### Translation

The original English version of the TEQ was translated into simplified Chinese according to standard guidelines that are widely accepted for the successful translation of measures in cross-cultural research. Two translators independently forward translated the original TEQ from English into simplified Chinese. The research team conducted two rounds of meetings to discuss the translation until they reached a consensus. Then, two native English speakers, who are also fluent in Chinese, were invited to back-translate the Chinese into English separately. Differences in the original and the back-translated versions were discussed until a consensus was reached by joint agreement of all translators and researchers.

### Participants

Medical students, from two universities in Guangzhou, were invited to participate in the study. The data were collected through a cross-sectional online survey from October to November in 2019. The officers in charge of student affairs from the two universities were approached first, and then, using WeChat (a multi-purpose messaging mobile application), all the students received a brief announcement as well as a link to an online questionnaire. The questionnaire included three sections. The first section was the introduction of the survey and a consent form. The second section included 10 items, which was used to collect the respondents’ demographic information. The last section was the cTEQ. Only when the students had completely gone through the consent form and agreed with it did the survey start. Finally, a convenient sample of 1296 students, from 33/34 provinces of China (demographic information presented in the Appendix), was collected. The sample size met the requirements of factor analysis and other psychometric evaluations (Floyd and Widaman, 1995; DeVellis, 2017).

### Materials

#### cTEQ

The cTEQ, which consists of 16 items, was used to measure empathy. All items were scored on a five-point Likert scale ranging from 0 = never to 4 = always. Items 2, 4, 7, 10, 11, 12, 14, and 15 are negatively worded and are reverse scored. All responses are summed to generate a total score out of 64; high scores indicate more empathy.

### Statistical Analysis

Two subsamples with 624 respondents for each were randomly generated. The first subsample was used for exploratory factor analysis (EFA) and psychometric analyses (CTT, IRT, and RMT). The second subsample was used for confirmatory factor analysis (CFA) to further evaluate the model fit. All analyses were conducted using R (R foundation, Austria). The R packages used in each psychometric method are listed in the following sections. For data quality, responses with less than 5% of missing data were considered as acceptable and included in the data analysis (DeVellis, 2017). The psychometric properties of the cTEQ were assessed based on CTT, IRT, and RMT separately.

The “*psych*,” “*irr*,” “*corrr*,” and *“lavaan”* packages were used to conduct CTT analysis. The following analyses were carried out:

(1) Reliability of scaling: Cronbach’s alpha (α) is a measure of internal consistency, where alpha > 0.7 was considered acceptable (DeVellis, 2017). The test–retest reliability (40 students were randomly selected to complete the cTEQ twice with a 2-week interval) was assessed using the intraclass correlation coefficient (ICC), with a value ≥ 0.3 considered as acceptable (DeVellis, 2017). Moreover, a number of other statistics, similar to α, were calculated to estimate the reliability of the cTEQ. They were the Rho coefficient, coefficient ωt (the amount of reliable variance in a scale), and coefficient ωh (the estimate of the general factor saturation of a scale) (Zinbarg et al., 2005). As for each of the indicators, a value ≥ 0.7 indicated acceptable reliability (Appendix).
(2) Evaluation of scaling: the mean score, standard deviation, median, and skewness of each item were reported as well as ceiling and floor effects (more than 15% choosing extreme options was considered to be unacceptable) (DeVellis, 2017). The item-total correlation, average inter-item correlation (examine the item redundancy), and alpha if an item was dropped were used to evaluate the inner relationships among items. The value of item-total correlation coefficient greater than 0.3 or the value of inter-item correlation coefficient smaller than 0.8 were deemed as acceptable (DeVellis, 2017). If the alpha for a dropped item became smaller than the alpha if this item was included, the item was retained.
(3) Structure of scaling: For EFA, the Kaiser-Meyer-Olkin (KMO, >0.6) measure and Bartlett’s test of sphericity (*p* < 0.05) were first adopted to test the suitability of the data for conducting EFA (Xu et al., 2018). The number of factors was determined by the criterion of “eigenvalue > 1.0.” For CFA, the Tucker Lewis index (TLI, >0.9), Comparative Fit Index (CFI, >0.9), Root Mean Square Error of Approximation (RMSEA, <0.08), and Standardized Root Mean Square Residual (SRMR, <0.08) were used to assess the model fit (Thompson, 2004). Akaike information criteria (AIC), and Bayesian information criteria (BIC) were also used to evaluate the model fit, which the lower values indicate a better fit (DeVellis, 2017).

The generalized partial credit model (GPCM), a polytomous model for ordinal items, was adopted to conduct IRT analysis. This means the statistics of discrimination and difficulty of each item were separately estimated (Linden and Hambleton, 1997). The package “*mirt*” was used to perform the IRT analyses. The following analyses were carried out:

(1) The option characteristic curves (OCCs) were presented to describe the relationship between a latent ability and the performance on an item of interest and check graphically whether the response pattern was reasonable.
(2) Adequacy of the model: the indicator, S-χ^2^, was used to test the fit of each item. An item with a non-significant S-χ^2^ value indicated fit under the model.
(3) Local dependency was evaluated by checking the discrimination of each item. If discrimination of the item was less than 4, the item met the requirement of local dependency.
(4) The item information curves (IICs) and test information curve (TIC) were graphically presented to indirectly assess the reliability of the scale. Based on the formula suggested by Nunnally (1994), reliability = 1 − (1/information), and as mentioned in the section on CTT, reliability greater than 0.7 was considered acceptable.
(5) Differential item functioning (DIF) was checked for possible item bias caused by responses from different subgroups in the sample followed by Monte Carlo simulated empirical criteria (Mair, 2018). The magnitude of DIF was evaluated by pseudo-*R*^2^ values (McFadden’s *R*^2^). An effect size less than 0.13 was defined as a negligible effect, one between 0.13 and 0.26 as moderate, and greater than 0.26 was considered large (Mair, 2018).

Rasch model theory was implemented to investigate whether the items fit the model well. The partial credit model (PCM), known as the adjacent category logit model, is a polytomous form of a Rasch model that was used to do the analyses (“*eRm*,” “irt,” and “*iarm*” package):

(1) Reliability: reliability in RMT was assessed by reporting the value of the person separation index (PSI), which is a similar statistic to α. A higher value of PSI indicates better reliability (PSI > 0.7 was considered acceptable). Local dependence was also confirmed based on using the Q3 statistics.
(2) Item fit: infit and outfit mean square (MNSQ) statistics were calculated to check whether the items fit the expected model. No gold standard exists to strictly define the acceptable range of the two MNSQ indicators. Based on Wright and Linacre’s (1994) suggestions, if the data fit the RM, the value of the two indicators should range between 0.6 and 1.4. The infit- and out-fit *t*-test were applied as well as the Bonferroni correction, where the *p*-value was set at <0.05/16 = 0.003 (Porta, 2014).
(3) A person-item map was obtained to graphically examine the distribution of the items and the person measures on the same continuum and obtain validity evidence of the questionnaire.

## Results

**FIGURE 1:**
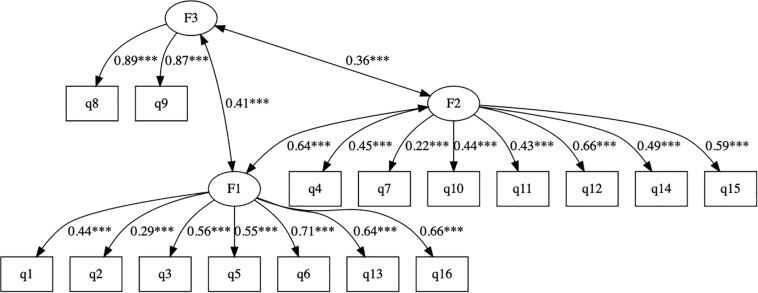
The CFA of the cTEQ with 3-factor structure. *p < 0.05, **p < 0.01, ***p < 0.001.

Using a test subsample, EFA suggested a three-factor structure of the cTEQ, with all 16 items having sufficient factor loadings (>0.3). The first factor explained 42% of the total variance. Item 7 did not perform well in the one- (0.18) and two-factor models (0.19). CFA (Figure 1) using another subsample confirmed that the three-factor model with satisfactory RMSEA (0.061), SMRM (0.061), TLI (0.88), and CFI (0.9) performed better than one- or two-factor models (the scree plot, and results of EFA and CFA are presented in the Appendix). The three factors consisted of items 1, 2, 3, 5, 6, 13, and 16 [F1: positive empathy], items 8 and 9 [F2: neutral empathy], and items 4, 7, 10, 11, 12, 14, and 15 [F3: negative empathy], respectively.

Table 1 presents the results of the CTT analyses. The overall α for the cTEQ was 0.81. The coefficients of inter-item correlation and item-total correlation coefficients are presented, and no item violated the criteria. Except for items 3, 4, 10, and 12, all of the other items had acceptable test–retest reliability. The mean score of the cTEQ items ranged between 2.1 (item 2) and 3.4 (item 15). Skewness for all items was negligible; Item 15 showed more severe skewness than the other items. Multiple items were flagged for evidence of ceiling effects, especially item 15, which reflected a very severe ceiling effect (% = 53.09).

**TABLE 1:** The results of CTT model in evaluating the psychometric property of cTEQ (*n* = 648).

	**Alpha^#^**	**Average inter-item correlation**	**Item-total correlation**	**Test–retest reliability**	**Mean**	**SD**	**Skewness**	**Floor effect**	**Ceiling effect**
Overall	0.81			0.78	42.28	6.62	–0.04		
Item 1	0.80	0.2	0.48	0.64	2.3	0.75	–0.13	1.70	5.25
Item 2	0.80	0.18	0.45	0.55	2.1	0.81	–0.19	3.55	2.78
Item 3	0.80	0.21	0.49	0.24	2.5	0.79	–0.29	0.93	9.10
Item 4	0.80	0.22	0.52	0.29	3.0	0.78	–0.78	0.46	27.16
Item 5	0.79	0.27	0.59	0.32	2.9	0.75	–0.52	0.62	21.45
Item 6	0.89	0.26	0.59	0.43	2.7	0.8	–0.39	1.23	14.81
Item 7	0.82	0.09	0.31	0.34	2.3	0.89	–0.36	3.24	6.94
Item 8	0.80	0.25	0.55	0.44	2.8	0.76	–0.39	0.77	14.51
Item 9	0.80	0.22	0.5	0.63	2.8	0.76	–0.34	0.46	16.67
Item 10	0.81	0.16	0.44	0.20	2.3	0.97	–0.16	3.55	10.34
Item 11	0.81	0.16	0.42	0.42	2.4	0.84	–0.21	1.54	8.33
Item 12	0.79	0.29	0.65	0.24	2.8	0.74	–0.65	0.62	15.12
Item 13	0.79	0.3	0.66	0.54	2.5	0.8	–0.07	0.62	8.49
Item 14	0.80	0.18	0.47	0.31	2.8	0.92	–0.93	2.62	17.44
Item 15	0.80	0.21	0.5	0.50	3.4	0.77	–1.23	0.46	53.09
Item 16	0.79	0.27	0.61	0.45	2.6	0.8	–0.15	0.93	13.12

*^#^For overall, the alpha represents the internal reliability of the scale; for each item, alpha represents the alpha if the item was dropped. SD, standard deviation; ICC, intraclass correlation coefficient.*

**FIGURE 2:**
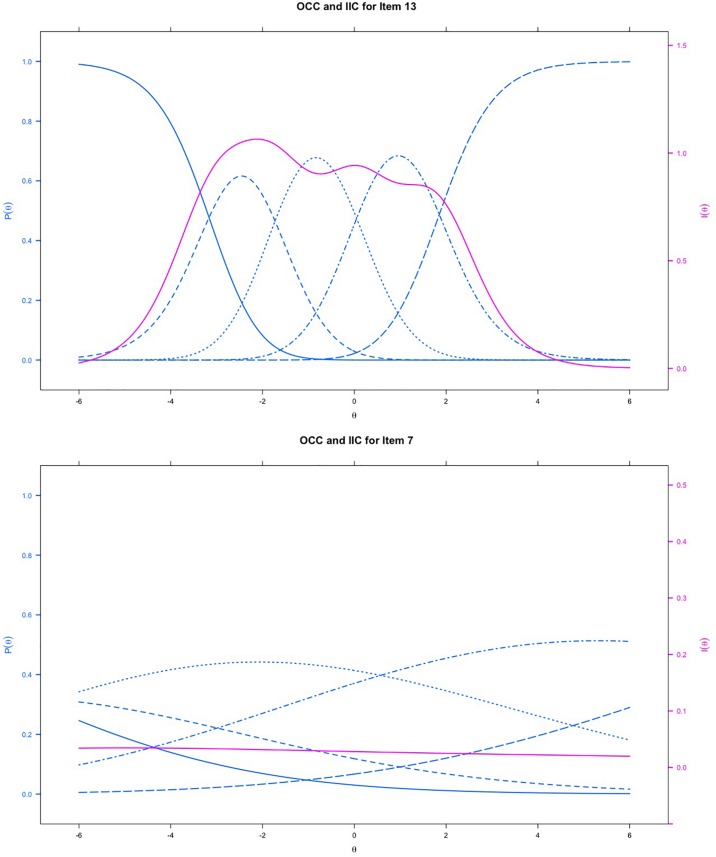
The item-category and item information curves of item 13 and item 7 based on IRT model; blue lines represents the OCC and red line represents the IIC.

**FIGURE 3:**
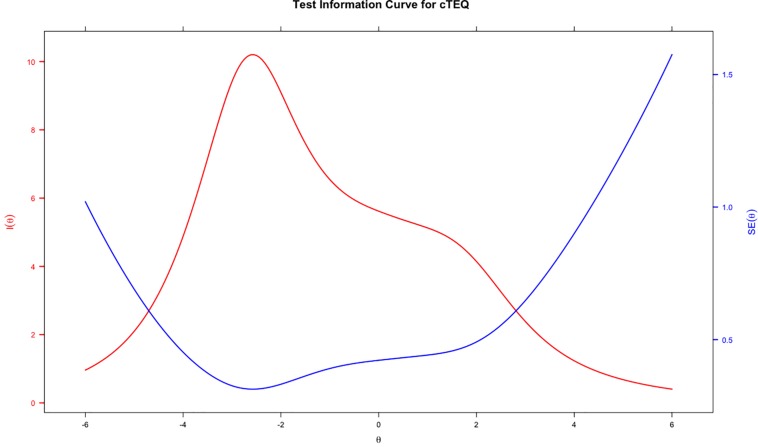
The test information curve of the cTEQ based on IRT model.

Table 2 indicates the results of IRT analyses based on GPCM. The discrimination value for all items ranged between 0.19 and 1.64, which indicated that no item might be sufficiently distinguishable to identify individuals with either low or high empathy. The significant *p*-value of S-χ^2^ statistics indicated that the majority of the items fit the scale, except for item 14 (significant), which possibly was misfitting. The OCCs and IIC were used to visualize the psychometric properties of the items. Examples of good (item 13) and bad items (item 7) are presented in Figure 2. The blue lines represent the OCCs and the pink line represents the IIC. For item 13, the OCCs followed the same expected order as the response categories and the IIC covered a large area, indicating that rich information was provided by this item. By contrast, the OCCs for item 7 were obviously disordered, and its IIC was very flat, which means very little information was provided by this item. The TIC of the cTEQ (Figure 3) indicated that 96.05% of the information was provided by the range of the latent trait (empathy) between −6 and 6. Further analysis indicated that the range between −6 and 0 provided 65.47% of the information and the range between 0 and 6 provided another 30.58% of the information. Two items were identified showing DIF on the sex variable. Among them, item 7 showed non-uniform DIF, whereas item 16 showed uniform DIF. However, the magnitude of DIF was negligible for both of them (Appendix).

**TABLE 2:** The results of IRT model in evaluating the psychometric property of cTEQ (*n* = 648).

	**Discrimination**	**Threshold indices**				**S-χ ^2^**	***p*-value**	**Test information (%, range)**		
	**a**	**b1**	**b2**	**b3**	**b4**			**−6 ∼ 6**	**−6 ∼ 0**	**0 ∼ 6**
Item 1	0.73	–2.89	–3.24	0.72	3.25	62.29	0.26	96.05	65.47	30.58
Item 2	0.54	–3.55	–2.43	1.72	4.74	79.02	0.07			
Item 3	0.8	–3.61	–2.76	–0.26	2.6	60.96	0.27			
Item 4	0.79	–3.96	–2.54	–2.03	1.14	69.86	0.05			
Item 5	1.36	–2.53	–2.88	–1.04	1.11	52.41	0.09			
Item 6	1.15	–2.28	–2.86	–0.48	1.59	43.16	0.59			
Item 7	0.19	–7.18	–6.55	0.57	8.96	59.39	0.76			
Item 8	1.06	–2.75	–2.99	–0.7	1.73	52.0	0.25			
Item 9	0.9	–3.46	–3.25	–0.9	1.71	73.56	0.01			
Item 10	0.37	–4.51	–2.86	0.73	3.39	84.07	0.16			
Item 11	0.41	–5.22	–3.91	0.29	4.15	89.94	0.02			
Item 12	1.3	–3.02	–2.31	–1.05	1.57	56.13	0.14			
Item 13	1.64	–3.17	–1.73	0.05	1.85	52.45	0.13			
Item 14	0.48	–3.08	–2.23	–2.46	2.68	146.3	0			
Item 15	0.7	–3.24	–3.5	–2.22	–0.57	58.74	0.056			
Item 16	1.26	–2.72	–2.53	–0.16	1.6	47.24	0.42			

*b, difficulty; b1 is the threshold between option 1 and 2; b2 is the threshold between option 2 and 3; b3 is the threshold between option 3 and 4; b4 is the threshold between option 4 and 5.*

**FIGURE 4:**
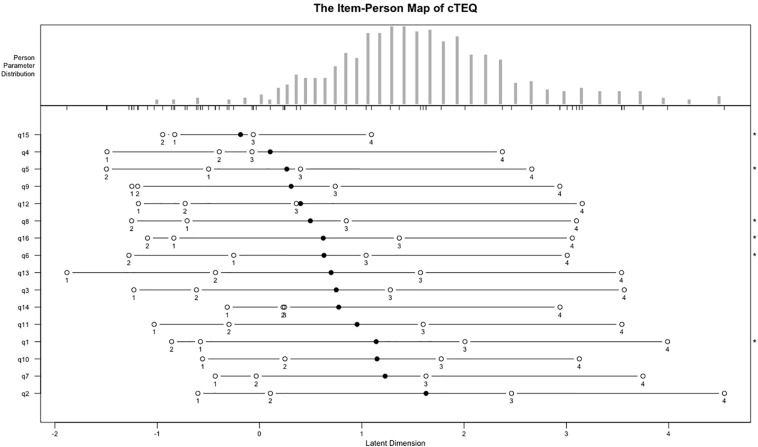
The item-person map of the cTEQ based on Rasch model.

Table 3 provides the results of RMT analyses. The chi-square test was significant for items 7, 10, 11, 12, 13, and 14, which suggested that they may not fit the model well. Item 7 had outfit MNSQ values outside of the range between 0.6 and 1.4, which suggested that it might be misfitting. The value of PSI was 0.83, which is considered acceptable. The item-person map indicated that the positions of the items was ordered alongside the latent scale but did not fit the distribution of respondents well. Some items showed overlap on the scale of latent trait to some extent (Figure 4).

**TABLE 3:** The results of Rasch model in evaluating the psychometric property of cTEQ (*n* = 648).

	**Outfit**	**Infit**	**Location**	**T1**	**T2**	**T3**	**T4**	**PSI**
Item 1	1.03	1.04	1.14	–0.58	–0.86	2.01	3.99	0.83
Item 2	1.13	1.1	1.63	–0.6	0.11	2.46	4.54	
Item 3	1.04	1.03	0.75	–1.23	–0.62	1.28	3.57	
Item 4	0.96	0.95	0.1	–1.49	–0.39	–0.07	2.37	
Item 5	0.84	0.87	0.27	–0.5	–1.5	0.4	2.66	
Item 6	0.9	0.89	0.63	–0.25	–1.28	1.04	3.01	
Item 7	1.42***	1.36***	1.23	–0.43	–0.03	1.63	3.75	
Item 8	0.91	0.93	0.5	–0.71	–1.25	0.85	3.1	
Item 9	1.01	0.99	0.31	–1.25	–1.19	0.74	2.94	
Item 10	1.27***	1.23**	1.15	–0.56	0.25	1.78	3.13	
Item 11	1.18*	1.15	0.95	–1.03	–0.3	1.6	3.54	
Item 12	0.78*	0.78*	0.4	–1.18	–0.73	0.36	3.16	
Item 13	0.79**	0.79**	0.7	–1.88	–0.43	1.57	3.54	
Item 14	1.34***	1.14	0.77	–0.32	0.23	0.24	2.94	
Item 15	0.91	0.96	–0.19	–0.83	–0.95	–0.06	1.09	
Item 16	0.86	0.86	0.62	–0.84	–1.09	1.37	3.06	

PSI, person separation index; T, threshold; T1, threshold between option 1 and 2; T2, threshold between option 2 and 3; T3, threshold between option 3 and 4; T4, threshold between option 4 and 5. *p < 0.05; **p < 0.01; ***p < 0.001.

Based on the psychometric analyses (CTT, IRT, and RMT), we found that (1) the items belonging to the last factor, especially, items 7 and 14, showed some problems on integral performance, and (2) F2 only had two items, which might be not theoretically reasonable. We, thus, further explored the structure of cTEQ using CFA based on different combinations of the items and factors. The CFA confirmed that cTEQ-14 (no items 7 and 14) items performed better than cTEQ-16 items (the results are presented in the Appendix).

## Discussion

This study introduced the cTEQ as a new measure to evaluate empathy in the Chinese population, and examined its psychometric properties based on three methods of CTT, IRT, and RMT in a single dataset. Compared with the reliability of the TEQ reported in other countries, for example, α of 0.85 in Canada (Spreng et al., 2009), α of 0.79 in Turkey (Totan et al., 2012), α of 0.72 in Greece (Kourmousi et al., 2017), and α of 0.79 in Korea (Kim and Han, 2016), the CTT analyses confirmed the good reliability of the cTEQ based on the test level, whereas the IRT and RMT analyses, provided more specific information to assess the performance of the cTEQ on the item level. Considering the findings from our analyses, a number of items need potential modification.

Considering the results of the CTT analyses, items 3, 4, 10, and 12, which tap the domains of sympathetic physiological arousal (item 3), feeling the same emotion as another (item 4), and frequency of behaviors demonstrating appropriate sensitivity (items 10 and 12), seemed to have some problems with responsiveness (low test–retest reliability). Three of them were negatively scored, which Lin et al. (2014) indicated might lead to low retest reliability. Serious concerns were raised about item 15 (“silly to cry for others”). It was the “easiest” item, with very negative skewness and had a severe ceiling effect. This finding was similar to that reported by Kourmousi et al. (2017) in Greek. Overall, items belonging to the domain of “frequency of behaviors demonstrating appropriate sensitivity” (items 10, 14, and 15) might have some problems compared to items from the other domains. It would be better to consider reporting their results as a separate score rather than combining them in the total score. Moreover, we found the cTEQ would have a higher alpha if item 7 (“steer the conversation”) was dropped, which indicated that removing it from the cTEQ should also be considered.

No previous TEQ validation study has adopted the methods of IRT or RMT in their analyses. Our study indicated that these methods provide rich item-level information for understanding the structure of the questionnaire. For the IRT analyses, the TIC confirmed that the cTEQ provided good information on the evaluation of the targeted latent trait. However, the OCC results showed that, although the cTEQ might be good at identifying people with low empathy, for individuals with high empathy, the instrument might not be very effective and sensitive. The OCCs, IICs, and TIC provided graphical evidence to assess the psychometric properties of the cTEQ. Overall, items 7, 14, and 15 showed moderate problems on the OCCs, in which the pattern of the curves did not fit the order of the responses. Additionally, the IICs confirmed that items 7, 10, and 11 provided very little information about the respondents’ latent trait in the test (the OCCs and IICs for cTEQ are provided in the Appendix). Furthermore, the significant *p*-value of S-χ^2^ for item 14 indicated that there might be some degree of misfit of this item. Overall, the items need several revisions, especially items 7 and 14.

As expected, the data performed worse on the stricter RMT than IRT model. Outfit and infit MSQN statistics showed a number of items might be insufficient to assess the targeted latent trait. The unexpected threshold ordering of items 14, 15, and 16 further suggested that modifications are needed. Moreover, the item-person map revealed, first, some items might assess a similar latent trait of empathy using the cTEQ, for example, items 6 and 16. They shared similar positions and overlapped on the continuum. However, for some other items, for example, items 2 and 7, the gap in the latent trait between them was larger than the others. This could lead to some problems when gaining information about an individual’s empathy using the cTEQ. Especially, in this study, we saw that a high proportion of respondents’ latent traits coincidently fell in the gap between items 2 and 7. Trevor and Fox suggested that a quick remedy is to find more appropriate persons to retest the questionnaire. However, we believe that other appropriate items might be developed to improve the precision (Bond, 2007). Another implication raised by the item-person map was that the difficulties of all the items only ranged between 0 and 2 (latent trait), and the majority of them (nearly 12/16) were targeted on the low side of the continuum (≤1). This result was graphically confirmed as well as by the TIC of the IRT analyses. The cTEQ might have some potential problems differentiating people with mid to high levels of empathy. However, the results from the international studies were conflicting. In the Greek study, 11/16 items had average scores ≥ 3.0 and five of them scored even higher than 3.5 (3.5/4) (Kourmousi et al., 2017). However, in the Turkish study, the mean overall score was only about 38/64, which was obviously lower than the other countries (Totan et al., 2012). Given that no previous studies have used IRT models to assess the validity of the TEQ, more investigations on other populations are needed.

Item invariance is one of the most important attributes for a good scale (DeVellis, 2017). To measure item invariance across the different sub-samples of the cTEQ, we set two variables, sex and ethnicity, as the anchor items to detect DIF in this study. The results showed that no item showed DIF on ethnicity, although two items, items 7 and 16, demonstrated minor non-uniform and uniform DIF, respectively, in terms of sex. For item 7, male respondents scored higher on the cTEQ than female respondents when respondents with the latent trait (empathy) ranged between −4 and −2.5. Furthermore, the trend then reverted when respondents’ latent trait moved to the range between −2.5 and 4. For item 16, male respondents always reported a higher score than female respondents. There are many possible reasons leading to DIF, for example, sample selection or survey administration (Nguyen et al., 2014). Given that our data for this study came from an online survey, all the respondents were relatively young people, and the magnitude of the DIF was negligible, we decided not to remove the items based on the results of DIF analyses. Further tests with different populations are therefore needed.

In this study, a three-factor model of cTEQ was confirmed. However, based on the analyses of CTT, IRT, and RMT, we found that there is a negative influence of items 7 and 14 on the psychometric performance of the cTEQ, and the two-factor model (no F2) showed a good fit as similar as the three-factor model when checking the different fit statistics. Considering the parsimony is one of the most important attributes of scale development and the cTEQ-10 (without F2 and items 7 and 14) showed an acceptable internal consistency (α = 0.79) using our sample, we should put the development of a short version of cTEQ on the schedule. Although CFA failed to confirm the original structure of the cTEQ, and some items did not perform well in our analyses, after fully considering the evidence, we decided to retain all 16 items in the cTEQ. A minor reason is that, even though IRT and RMT revealed some concerns about the items, CTT confirmed that the overall performance of the 16-item cTEQ is acceptable. The major reason is that the representativeness of the sample is a limitation of our study. The TEQ was not specifically designed for assessing empathy of medical university students. Our young, healthy, and highly educated sample might pose some positive or negative biases that influenced evaluation of their empathy. Other limitations included that we did not evaluate the convergent validity of the cTEQ. Empathy should have some relationships with mental health, cognitive ability, and so on. A questionnaire that evaluates other emotional functions might provide more information relevant to the assessment of the psychometric properties of the cTEQ. Thus, in future studies, other subgroup populations and relative instruments should be included. The other limitation is that no external validation data were provided for the cTEQ scales, particularly using criteria that are especially culturally relevant, which should be further explored in the future.

In the medical literature, adopting three different psychometric methods in a single project to evaluate the quality of measurement is limited (Kean et al., 2018). IRT and RMT can go beyond the test information provided by CTT and provide more information about items on potential causes and areas for improvement (Petrillo et al., 2015). Although compared with CTT, the results of IRT and RMT analysis are less straightforward, it can improve the precision of measurement (Fries et al., 2005). For example, in this study, the TIC and the item-person map indicated that the ability of the cTEQ might be insufficient to differentiate individuals with moderate to high empathy. However, given that this is the first study to adopt IRT methods to assess the TEQ worldwide, we need to report the findings with caution.

## Conclusion

This study reported that the cTEQ is a parsimonious and useful instrument to evaluate medical students’ empathy in China. However, CFA, IRT, and RMT analyses suggested that the structure and items of the cTEQ might need some modifications. Our study supported a three-factor structure cTEQ with 14 items performed better than the original version; however, given that the TEQ was designed to assess empathy in the general population, the psychometric quality and performance of the cTEQ needs to be further measured in other Chinese general populations.

## Data Availability Statement

The raw data supporting the conclusions of this article will be made available by the authors, without undue reservation, to any qualified researcher.

## Ethics Statement

The study was approved by the institutional review board of the third affiliated hospital of Southern Medical University (Ethical approval ID: 2019-044). All participants provided informed consent.

## Author Contributions

DW and RX conceptualized the study. RX conducted the data analysis and prepared the manuscript. EW and SL contributed to the translation and data analysis. JC and LZ were responsible for the data collection. DW supervised the whole study. All authors have read the manuscript and approved the submission.

## Conflict of Interest

The authors declare that the research was conducted in the absence of any commercial or financial relationships that could be construed as a potential conflict of interest.
