# Supplementary material for: Validation of the Toronto Empathy Questionnaire (TEQ) Among Medical Students in China: Analyses Using Three Psychometric Methods
Source: Front Psychol. 2020 Apr 28;11:810. doi: 10.3389/fpsyg.2020.00810 (PMC7199516; doi:10.3389/fpsyg.2020.00810)
Supplement: Supplementary file 1 [file Data_Sheet_1.docx]

**Appendix**

Table A-1Demographics of the sample (n=1296)

|  | n | % | cTEQ score  (0-64) | Sd |
| --- | --- | --- | --- | --- |
| Sex |  |  |  |  |
| Male | 321 | 24.8 | 41.1 | 7.09 |
| Female | 975 | 75.2 | 42.7 | 6.29 |
| Age in years, mean (sd) | 19.09 (1.92) |  |  |  |
| Ethnicity |  |  |  |  |
| Han | 1141 | 88.0 | 42.3 | 6.44 |
| Others | 155 | 12.0 | 42.3 | 7.22 |
| Educational level |  |  |  |  |
| Undergraduate | 1245 | 96.1 | 42.3 | 6.57 |
| Postgraduate | 51 | 3.9 | 41.6 | 5.60 |
| Year in school |  |  |  |  |
| 1 | 456 | 35.2 | 42.4 | 6.75 |
| 2 | 276 | 21.3 | 42.1 | 6.83 |
| 3 | 368 | 28.4 | 42.9 | 6.07 |
| 4 | 178 | 13.7 | 41.1 | 6.19 |
| 5 | 18 | 1.4 | 40.4 | 7.36 |
| Health status (0-10), mean (sd) | 7.34 (1.52) |  |  |  |
| Number of brothers or sisters |  |  |  |  |
| 0 | 452 | 34.9 | 41.7 | 7.17 |
| 1 | 518 | 40.0 | 42.8 | 6.17 |
| 2 | 226 | 17.4 | 42.4 | 5.83 |
| ≥3 | 100 | 7.7 | 42.0 | 6.67 |
| Province of residence |  |  |  |  |
| Guangdong | 505 | 38.97 |  |  |
| Anhui | 65 | 5.02 |  |  |
| Hunan | 62 | 4.78 |  |  |
| Inner Mongolia | 50 | 3.86 |  |  |
| Henan | 44 | 3.4 |  |  |
| Jiangxi | 42 | 3.24 |  |  |
| Guizhou | 41 | 3.16 |  |  |
| Hubei | 40 | 3.09 |  |  |
| Fujian | 39 | 3.01 |  |  |
| Guangxi | 39 | 3.01 |  |  |
| Sichuan | 37 | 2.85 |  |  |
| Gansu | 35 | 2.7 |  |  |
| Shanxi | 28 | 2.16 |  |  |
| Liaoning | 26 | 2.01 |  |  |
| Shandong | 26 | 2.01 |  |  |
| Jiangsu | 23 | 1.77 |  |  |
| Yunnan | 19 | 1.47 |  |  |
| Xinjiang | 18 | 1.39 |  |  |
| Hainan | 17 | 1.31 |  |  |
| Hebei | 17 | 1.31 |  |  |
| Zhejiang | 17 | 1.31 |  |  |
| Chongqing | 16 | 1.23 |  |  |
| Qinghai | 16 | 1.23 |  |  |
| Jilin | 13 | 1 |  |  |
| Heilongjiang | 12 | 0.93 |  |  |
| Shanxi | 12 | 0.93 |  |  |
| Beijing | 10 | 0.77 |  |  |
| Tianjin | 10 | 0.77 |  |  |
| Tibet | 7 | 0.54 |  |  |
| Ningxia | 4 | 0.31 |  |  |
| Macao | 3 | 0.23 |  |  |
| Shanghai | 2 | 0.15 |  |  |
| Hong Kong | 1 | 0.08 |  |  |

Sd, standard deviation;


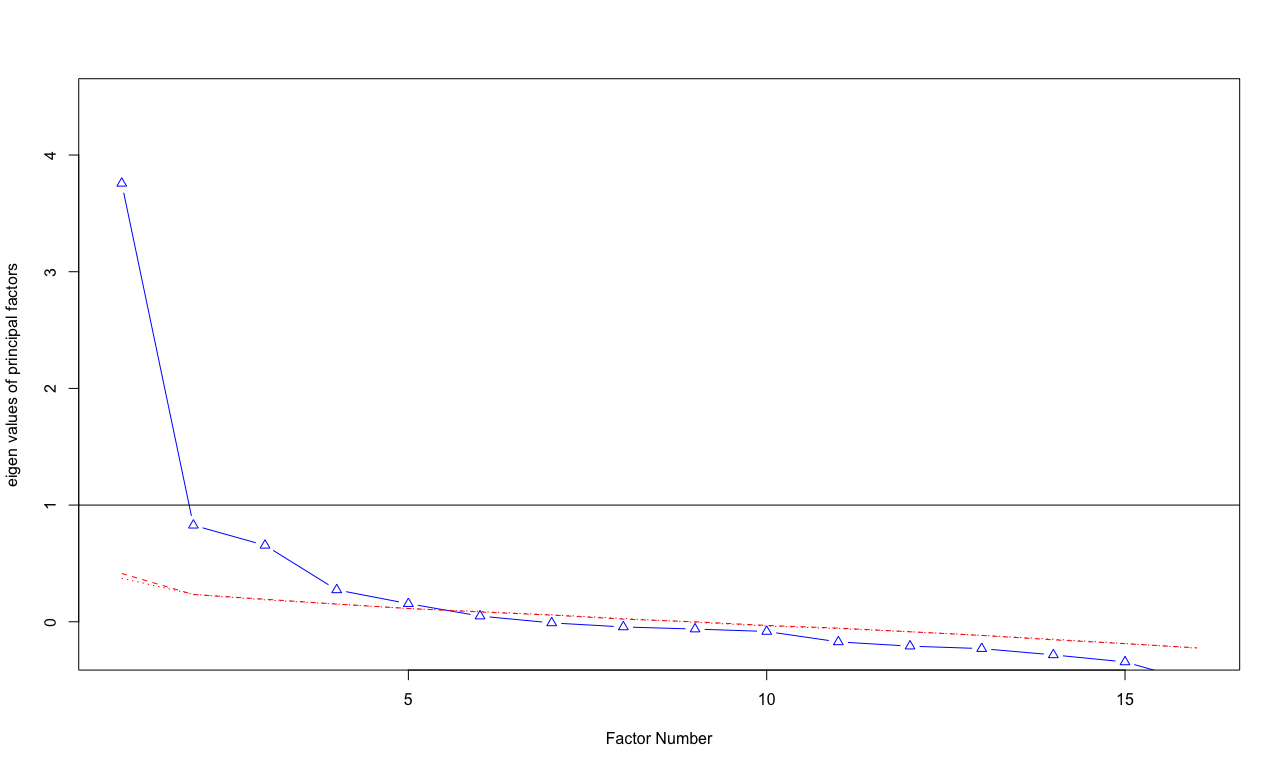


Figure A-1 the Scree plot of cTEQ

Table A-2 The results of EFA

| **cTEQ** | **Factor loadings** | | | | | |
| --- | --- | --- | --- | --- | --- | --- |
|  | **1-factor** | **2-factor** | | **3-factor** | | |
| Item 1 | 0.45 | 0.41 |  | 0.45 |  |  |
| Item 2 | 0.37 | 0.43 |  | 0.37 |  |  |
| Item 3 | 0.46 | 0.38 |  | 0.65 |  |  |
| Item 4 | 0.47 | 0.5 |  |  |  | 0.31 |
| Item 5 | 0.61 | 0.45 |  | 0.49 |  |  |
| Item 6 | 0.58 | 0.53 |  | 0.67 |  |  |
| Item 7 | 0.17 | 0.2 |  |  |  | 0.38 |
| Item 8 | 0.54 |  | 0.83 |  | 0.97 |  |
| Item 9 | 0.49 |  | 0.85 |  | 0.79 |  |
| Item 10 | 0.32 | 0.45 |  |  |  | 0.46 |
| Item 11 | 0.3 | 0.35 |  |  |  | 0.58 |
| Item 12 | 0.61 | 0.64 |  |  |  | 0.58 |
| Item 13 | 0.67 | 0.59 |  | 0.56 |  |  |
| Item 14 | 0.37 | 0.54 |  |  |  | 0.44 |
| Item 15 | 0.43 | 0.5 |  |  |  | 0.43 |
| Item 16 | 0.61 | 0.54 |  | 0.56 |  |  |

Table A-3 The results of CFA based on testing subpopulation

|  | RMSEA | TLI | CFI | SRMR | AIC | BIC |
| --- | --- | --- | --- | --- | --- | --- |
| Model 1 (1-factor model) | 0.114 | 0.578 | 0.6534 | 0.084 | 23479.39 | 23622.55 |
| Model 2 (2-factor model) | 0.078 | 0.803 | 0.831 | 0.066 | 23010.05 | 23157.69 |
| Model 3 (3-factor model) | 0.061 | 0.88 | 0.9 | 0.061 | 22851.09 | 23007.67 |
| Model 4 (3-factor without item 7) | 0.059 | 0.898 | 0.915 | 0.051 | 21169.77 | 21317.4 |
| Model 5 (3-factor without item 14) | 0.061 | 0.885 | 0.905 | 0.055 | 21262.96 | 21410.6 |
| Model 6 (3-factor without item 7 & 14) | 0.059 | 0.906 | 0.924 | 0.053 | 19576.14 | 19714.83 |
| Model 7 (2-factor [without F2]) | 0.065 | 0.854 | 0.878 | 0.055 | 20580.62 | 21710.37 |
| Model 8 (2-factor [without F2 and item 7]) | 0.062 | 0.881 | 0.902 | 0.049 | 18899.26 | 19020.05 |
| Model 9 (2-factor [without F2 and item 14]) | 0.065 | 0.861 | 0.886 | 0.055 | 18899.51 | 19112.31 |
| Model 10 (2-factor [without F2 and item 7 & 14]) | 0.061 | 0.893 | 0.914 | 0.047 | 17304.58 | 17416.42 |

Table A-4 the other indices of reliability of cTEQ

|  | Rho coefficient | Guttman’s Lambda 2 | McDonald’s ω_t_ | McDonald’s ω_h_ |
| --- | --- | --- | --- | --- |
| Value | 0.81 | 0.81 | 0.85 | 0.56 |


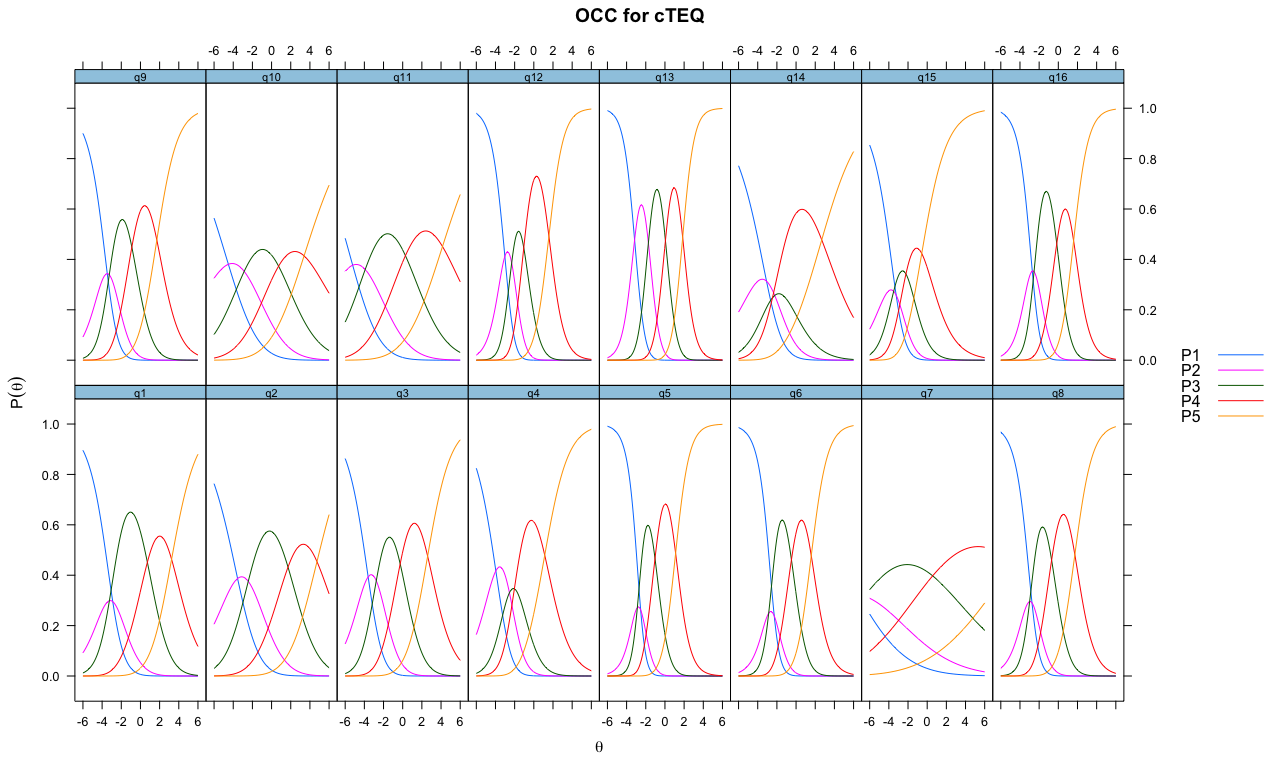


Figure A-2 the OCC for all the items of cTEQ


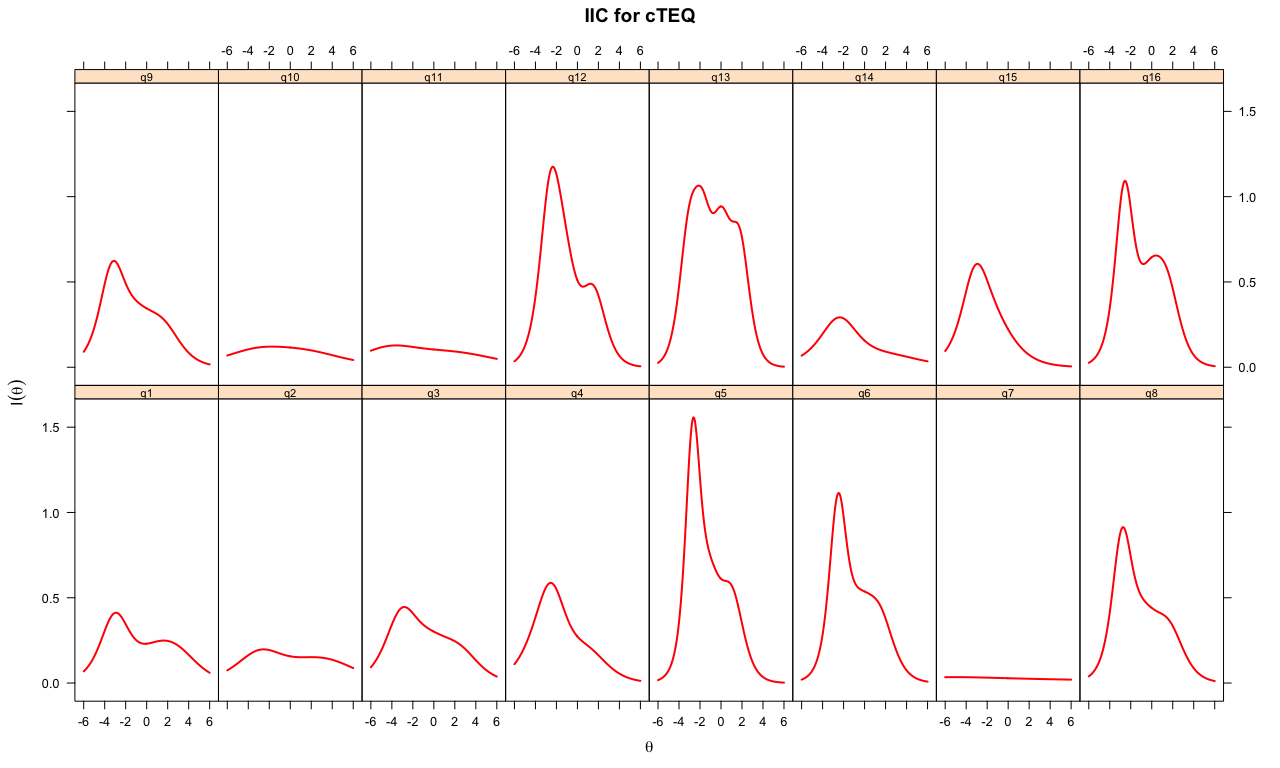


Figure A-3 the IIC for all the items of cTEQ


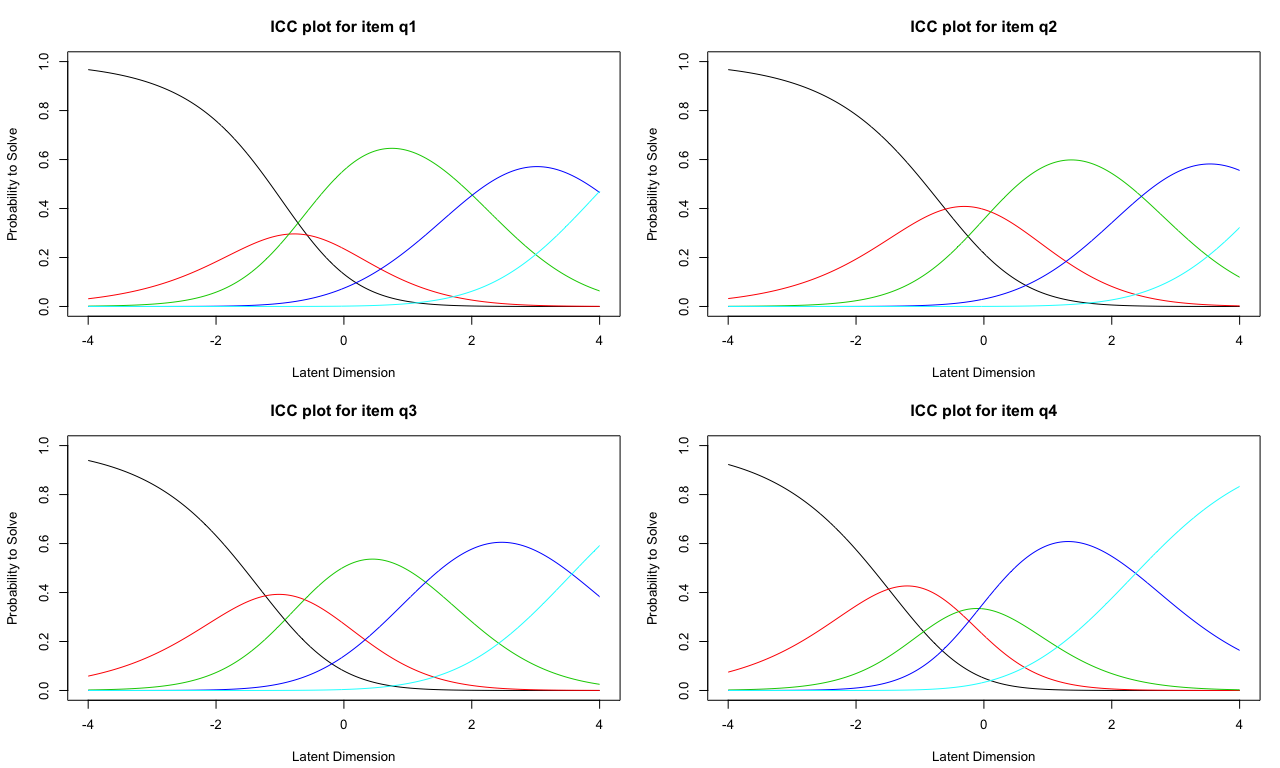


Figure A-4 the ICC plot for Item 1-4 based on Rasch analysis


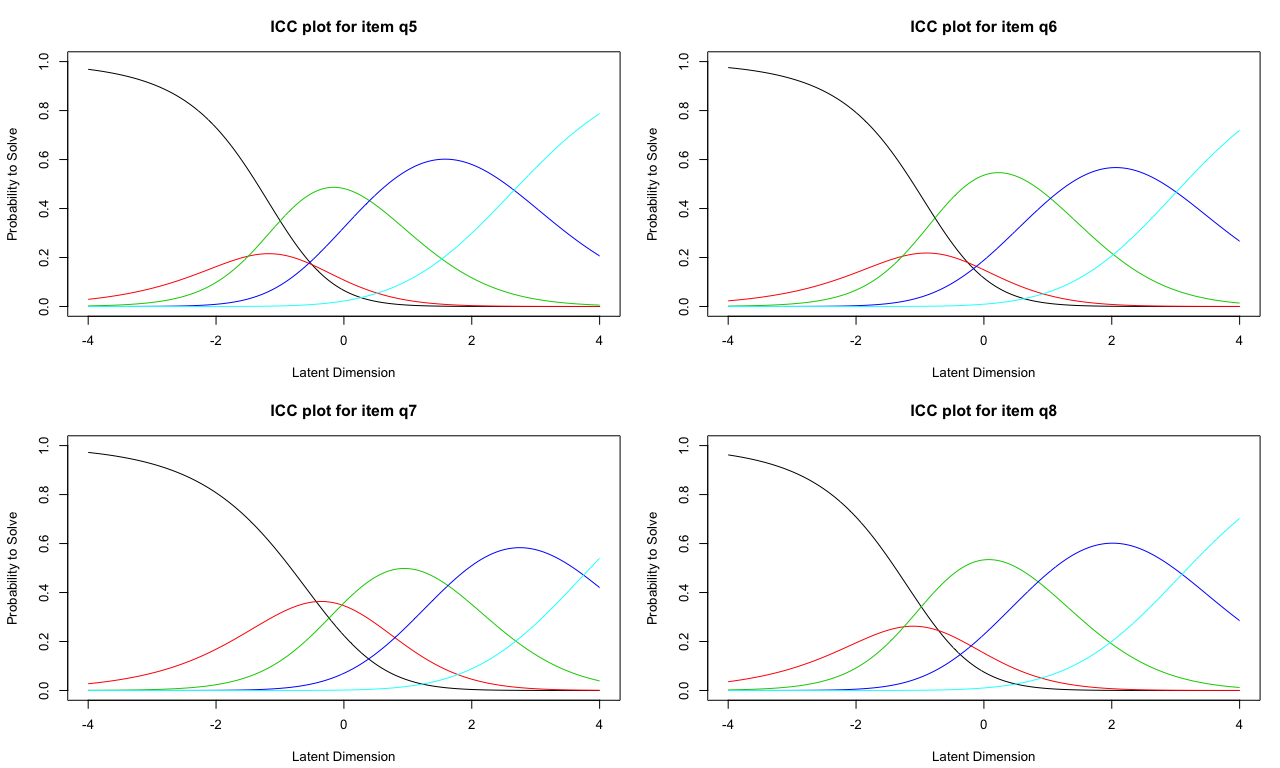


Figure A-5 the ICC plot for Item 5-8 based on Rasch analysis


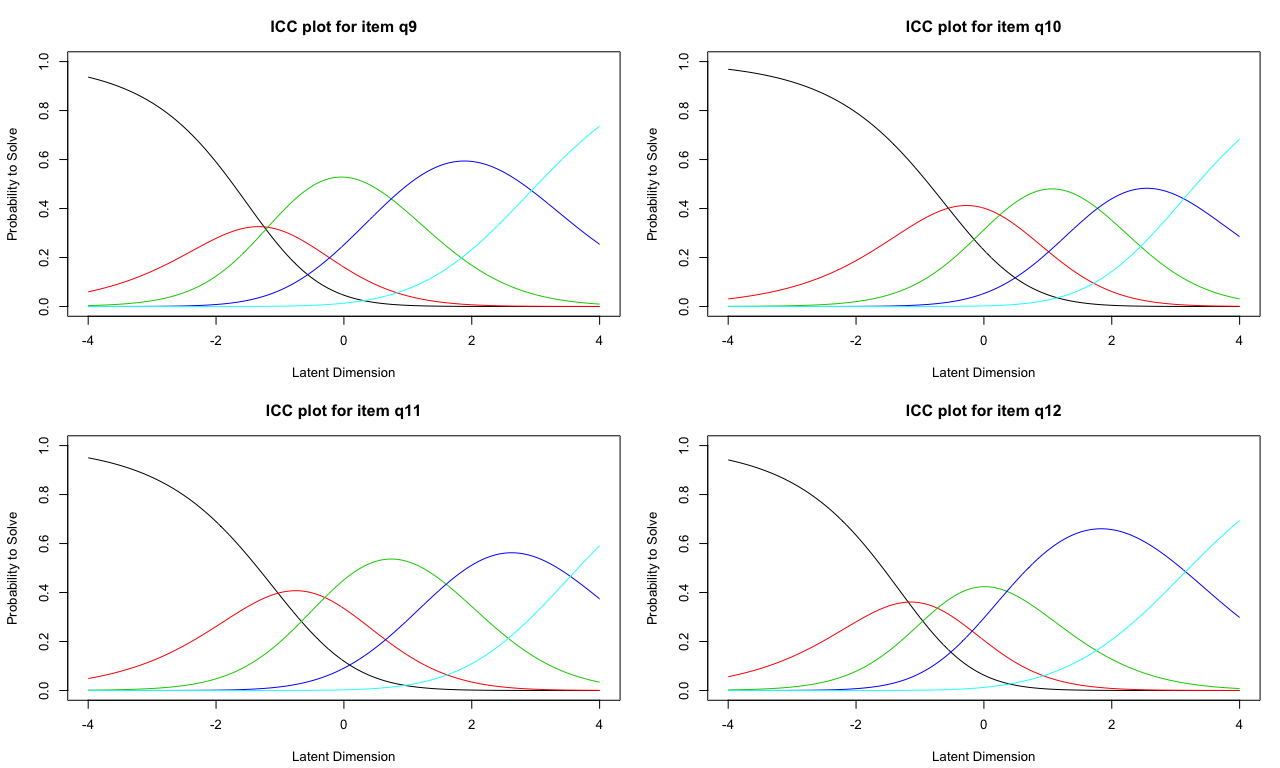


Figure A-6 the ICC plot for Item 9-12 based on Rasch analysis


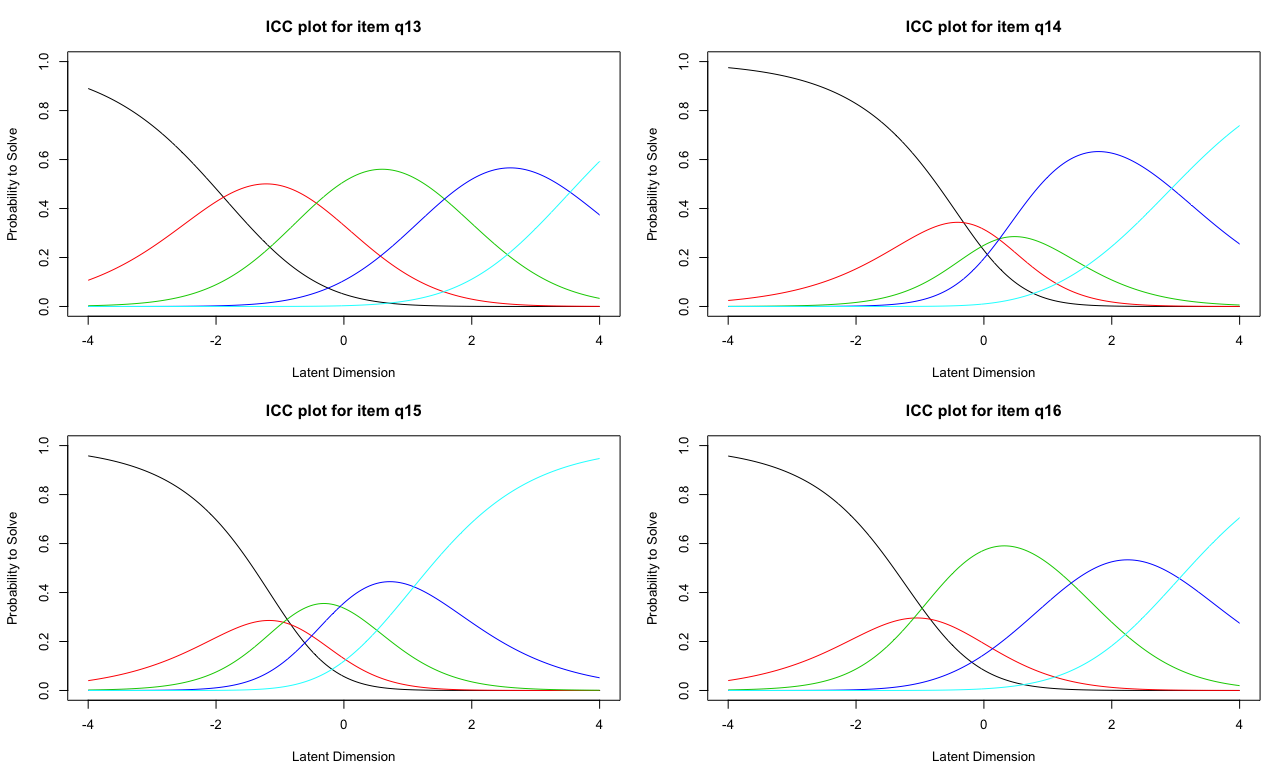


Figure A-7 the ICC plot for Item 13-16 based on Rasch analysis

**
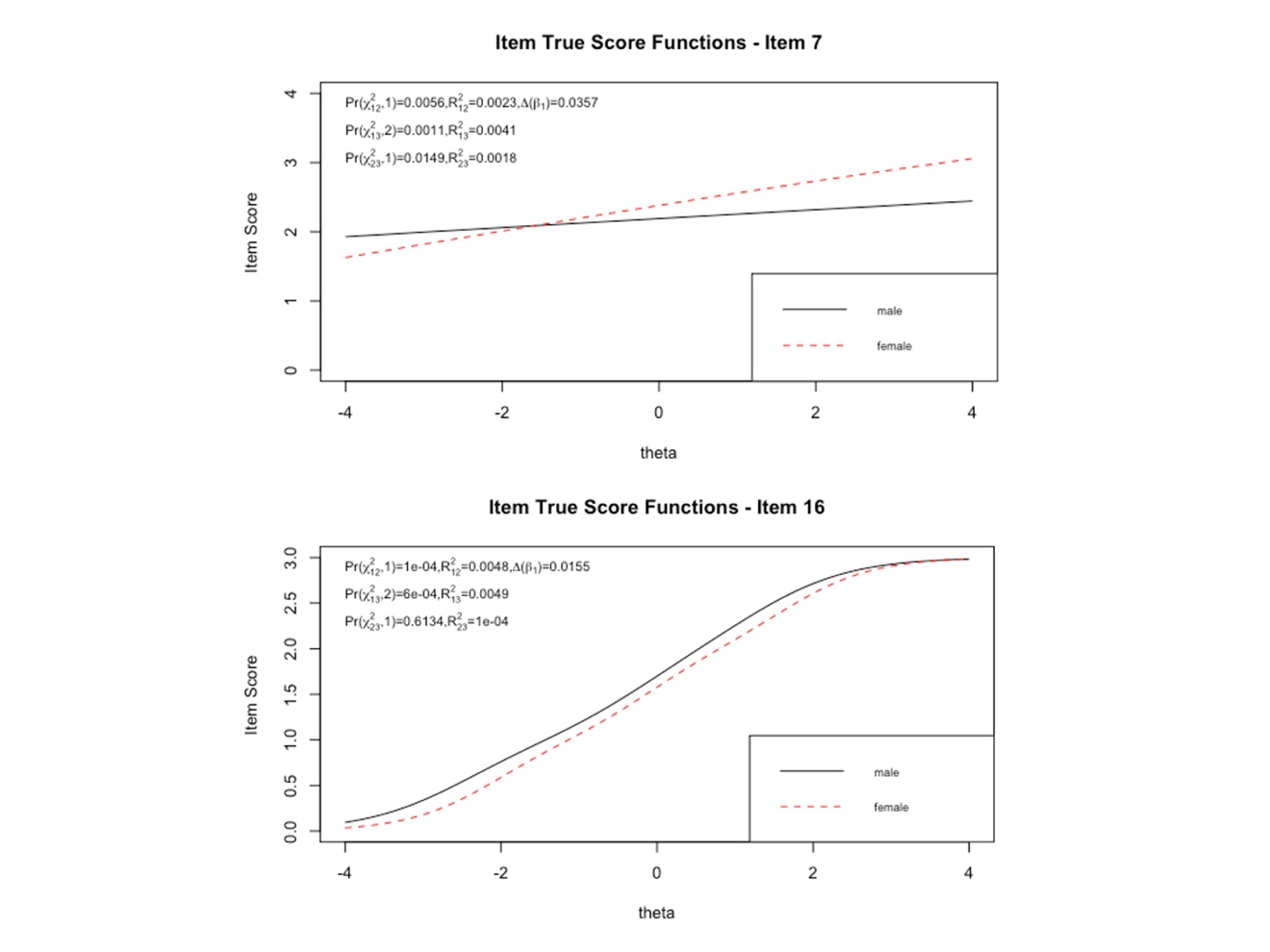
**

Figure A-8 the DIF items of cTEQ

cTEQ

请您仔细阅读下面的条目，并根据您感受和经历这些情况的程度，在相应的选项上进行勾选。所有的答案都没有正确或错误之分，请您尽可能的诚实回答所有的题目

|  |  | 从来  没有 | 极少 | 有时 | 经常 | 总是 |
| --- | --- | --- | --- | --- | --- | --- |
| 1 | 当别人为某事感到很兴奋的时候，我往往也觉得很兴奋 |  |  |  |  |  |
| 2 | 别人的不幸不会影响我太多 |  |  |  |  |  |
| 3 | 当别人受到了不尊重的对待，我往往会感到不安 |  |  |  |  |  |
| 4 | 即便是与我很亲近的人，我也不会为他/她的开心而感到开心 |  |  |  |  |  |
| 5 | 我乐于让他人感觉更愉悦。 |  |  |  |  |  |
| 6 | 我会同情和担忧比我不幸的人。 |  |  |  |  |  |
| 7 | 当一个朋友向我谈及一些不如意的事情时，我会试图扭转话题。 |  |  |  |  |  |
| 8 | 即使别人不说，我也能察觉他/她低落的情绪。 |  |  |  |  |  |
| 9 | 我觉得我通常可以察觉到别人情绪的变化 |  |  |  |  |  |
| 10 | 我不太同情那些因为自己的原因而造成严重疾病的人 |  |  |  |  |  |
| 11 | 当别人开始哭泣的时候，我会变得烦躁 |  |  |  |  |  |
| 12 | 我并不特别关心别人内心的感受 |  |  |  |  |  |
| 13 | 当我发现有人不安的时候，我会迫切想要提供帮助。 |  |  |  |  |  |
| 14 | 当我发现有人被不公正对待的时候，我不会对他们有很大的同情。 |  |  |  |  |  |
| 15 | 我觉得那些因为喜悦而流泪的人很傻 |  |  |  |  |  |
| 16 | 當我發現有人被佔便宜，我會有些想要保護他/她 |  |  |  |  |  |
